# Cancer Care Delivery and Women’s Health: The Role of Patient Navigation

**DOI:** 10.3389/fonc.2016.00002

**Published:** 2016-01-28

**Authors:** Jessica L. Krok-Schoen, Jill M. Oliveri, Electra D. Paskett

**Affiliations:** ^1^Comprehensive Cancer Center, The Ohio State University, Columbus, OH, USA; ^2^Division of Cancer Prevention and Control, Department of Internal Medicine, College of Medicine, The Ohio State University, Columbus, OH, USA; ^3^Division of Epidemiology, College of Public Health, The Ohio State University, Columbus, OH, USA

**Keywords:** patient navigation, breast cancer, cervical cancer, gynecological cancers, women’s health, cancer disparities

## Abstract

**Background:**

Patient navigation (PN) is a patient-centered health-care service delivery model that assists individuals, particularly the medically underserved, in overcoming barriers (e.g., personal, logistical, and system) to care across the cancer care continuum. In 2012, the American College of Surgeons Commission on Cancer (CoC) announced that health-care facilities seeking CoC-accreditation must have PN processes in place starting January 1, 2015. The CoC mandate, in light of the recent findings from centers within the Patient Navigation Research Program and the influx of PN interventions, warrants the present literature review.

**Methods:**

PubMed and Medline were searched for studies published from January 2010 to October 2015, particularly those recent articles within the past 2 years, addressing PN for breast and gynecological cancers, and written in English. Search terms included patient navigation, navigation, navigator, cancer screening, clinical trials, cancer patient, cancer survivor, breast cancer, gynecological cancer, ovarian cancer, uterine cancer, vaginal cancer, and vulvar cancer.

**Results:**

Consistent with prior reviews, PN was shown to be effective in helping women who receive cancer screenings, receive more timely diagnostic resolution after a breast and cervical cancer screening abnormality, initiate treatment sooner, receive proper treatment, and improve quality of life after cancer diagnosis. However, several limitations were observed. The majority of PN interventions focused on cancer screening and diagnostic resolution for breast cancer. As observed in prior reviews, methodological rigor (e.g., randomized controlled trial design) was lacking.

**Conclusion:**

Future research opportunities include testing PN interventions in the post-treatment settings and among gynecological cancer patient populations, age-related barriers to effective PN, and collaborative efforts between community health workers and patient navigators as care goes across segments of the cancer control continuum. As PN programs continue to develop and become a standard of care, further research will be required to determine the effectiveness of cancer PN across the cancer care continuum, and in different patient populations.

## Introduction

Profound advances in cancer screening, reductions in the prevalence of risk factors, and development of more effective treatments have positively contributed to increased longevity and quality of life among cancer survivors. Despite these improvements, disparities by race/ethnicity and socioeconomic status remain in cancer prevention, incidence, treatment, and mortality (1, 2). One approach to reduce cancer disparities is through patient navigation (PN). PN is a patient-centered, health-care service delivery model that assists individuals, particularly the medically underserved, in overcoming barriers to care (e.g., personal, logistical, system) across the cancer care continuum. PN is “navigation” in the health-care system compared to outreach, which is in the community and can use lay health advisors, community health workers (CHWs), etc. Typically, CHWs are trusted community members who provide information, support, and encouragement to receive screening tests (3). Previous intervention studies using CHWs to promote cancer screening have reported significantly increased screening rates for breast and cervical cancer (3, 4). CHWs and patient navigators differ in that CHWs work in the community, and their role ends when the patient enters the health-care facility. Patient navigators are typically housed in clinics; however, both the CHW and patient navigator complement each other in that they serve as a bridge between the health-care system and members of underserved communities.

Patient navigation began in 1990 by Harold Freeman who developed a PN program within a public hospital in Harlem, NY, USA to provide assistance to low-income women in need of breast cancer screening and timely follow-up to reduce diagnoses of late-stage breast cancer (5). Due to the promising results from Freeman’s initial PN program and continued evidence of effectiveness, PN has grown nationally as a standard of care (6, 7). PN studies have demonstrated that PN can improve rates of cancer screening (8, 9), ensure follow-up rates after an abnormal screening test (10, 11), and improve cancer care outcomes (i.e., time to treatment, quality of life) (12, 13).

Several recent literature reviews including Robinson-White et al. (14), Paskett et al. (15), and Wells et al. (16) have described the evolution of PN as a model to address cancer disparities. In 2011, a literature review by Paskett et al. (15) provided an update to the 2008 review by Wells et al. (16) on the efficacy of PN for cancer care. Paskett et al. (15) found that within the 3-year window since Wells’ review, the quantity of work in cancer PN literature was comparable to that of the previous years combined. In both reviews, most studies provided evidence for the effectiveness of increasing cancer screening rates including breast and gynecological cancers (15, 16). However, a paucity of research focusing on PN among cancer survivors during and after primary treatment and methodological limitations (i.e., lack of rigorous study design) of PN interventions were noted.

Since the 2011 literature review, many additional PN programs have been implemented especially those within the Patient Navigation Research Program (PNRP), funded by the National Cancer Institute (17) with support from the American Cancer Society (ACS). Active from 2005 to 2010, the PNRP provided funding to 10 institutions nationwide to develop and test interventions for follow-up and the initiation of treatment for four cancers for approved, validated screening tests: breast, cervical, prostate, and colorectal (18). Although any individual could benefit from PN, the PNRP focused efforts to identify and address barriers to care among populations experiencing cancer health disparities.

Breast and gynecological cancers are an optimal arena to use PN because of the known survival benefit of early detection through mammography and Pap tests with prompt follow-up of detected abnormalities. PN is particularly important in women’s cancers because of documented racial and ethnic disparities in cancer care across disease trajectories. In 2015, cervical cancer incidence rates among Hispanic women were the highest of any racial/ethnic group, 50% higher than those among non-Hispanic whites (19). In addition, the death rate for cervical cancer in black women was double than that in non-Hispanic white women (2.0 vs. 4.2 per 100,000, respectively) (19). It is notable that although white women had the highest breast cancer incidence rate, black women had the highest breast cancer mortality rate (19, 20). Thus, PN may be a strategy to help reduce these documented disparities.

The purpose of this review is to: (a) provide a summary of the recent literature (2010–2015) on PN and breast and gynecologic cancers from screening through treatment along the cancer care continuum; and (b) highlight research challenges and opportunities of PN that impact women’s health.

## Methods

PubMed and Medline were searched for studies published from January 2010 to October 2015, particularly those recent articles within the past 2 years, addressing PN for breast and gynecological cancers and written in English. Only original studies reporting quantitative, qualitative, or mixed methods results regarding PN that dealt with cancer screening, diagnosis, treatment, clinical trials, or survivorship were included in this review. Editorials, abstracts, anecdotal reports, literature reviews, and articles lacking data from original research were excluded, as were articles that included non-breast/non-gynecological cancers and/or men in the analyses. Search terms included patient navigation, navigation, navigator, cancer screening, clinical trials, cancer patient, cancer survivor, breast cancer, gynecological cancer, ovarian cancer, uterine cancer, vaginal cancer, and vulvar cancer.

A total of 209 articles referencing PN in women’s cancers were found, of which 180 did not meet the inclusion criteria, resulting in 29 articles that met the criteria for inclusion in this review. Several notable PNRP articles were excluded because they included non-breast/non-gynecological cancer and men in their analyses (21–25). The 29 articles were then divided into categories along the cancer care continuum (screening, diagnostic resolution, and after primary diagnosis). The articles were reviewed and summarized by one study author (Jessica L. Krok-Schoen). Questions regarding inclusion were resolved by consensus among the other two authors (Jill M. Oliveri and Electra D. Paskett). Each article was reviewed, and the results presented are organized by placement along the cancer care continuum described above. Table 1 comprises a summary of published cancer PN studies (*N* = 29).

**Table 1:** **Summary of published cancer patient navigation studies: 2010–2015**.

Reference	Cancer	Design	Participants	Results
**PATIENT NAVIGATION FOR CANCER SCREENINGS**				
Burhansstipanov et al. (26)	Breast	Natural experiment then a quasi-control study	313 African American, Latina, Native American, and poor White women who had not received a mammography in more than 18 months enrolled in a navigation intervention	Navigation improved mammography among women for all racial/ethnic groups who received the navigation intervention compared to those women in the non-navigated group
Marshall et al. (27)	Breast	Randomized controlled trial	1,358 African American female Medicare beneficiaries who were ≥65 years of age randomized to receive either patient navigation and educational materials (*n* = 638) or educational materials only (*n* = 720)	Women in the intervention group had significantly higher odds of being up to date on mammography screening compared to women in the education only group (OR = 2.26, 95% CI = 1.59–3.22)
Percac-Lima et al. (28)	Breast	Quasi-experimental intervention	91 Serbo-Croatian speaking women overdue or never had a mammogram who received individually tailored interventions to encourage breast cancer screenings	At baseline, 44.0% of women had a mammogram within the previous year, with the proportion significantly increasing to 67.0% after 1 year (*p* < 0.001)
Percac-Lima et al. (29)	Breast	Quasi-experimental intervention	188 refugee women eligible for breast cancer screening at an urban community health center. The comparison group was English (*n* = 2,072) or Spanish-speaking (*n* = 2,014) women eligible for breast cancer screening	Patient navigation increased screening rates in both younger and older refugee women (64.1% before intervention, 81.2% after intervention) and were similar to the English (80.0%) and Spanish-speaking women (87.6%)
Phillips et al. (30)	Breast	Controlled cluster randomized trial	3,895 inner city women were randomized to a phone-based navigation intervention (*n* = 1,817) and usual care (*n* = 2,078) groups	At baseline, there was no difference in mammography adherence between the usual care and intervention groups. After the 9-month intervention, mammogram adherence was significantly higher in the intervention group (87.0%) compared with the usual care group (76.0%) (*p* < 0.001)
Wang et al. (31)	Cervical	Two-arm, quasi-experimental pilot study	Chinese women (*n* = 134) who has not had a Pap test within the previous 12 months assigned to either patient navigation (education and navigation services) (*n* = 80) and control (education only) (*n* = 54) groups	In the 12 months following the program, Pap screening rates were significantly higher in the intervention group (70.0%) compared to the control group (11.1%) (*p* < 0.001)
**PATIENT NAVIGATION FOR DIAGNOSTIC RESOLUTION**				
Basu et al. (32)	Breast	Pre-post design, quasi-experimental intervention	176 women diagnosed with breast cancer enrolled in a nurse navigation program to increase timeliness to diagnostic resolution and consultation	Navigation was found to significantly shorten time to consultation for women older than 60 years but not for women 31-60 years of age
Battaglia et al. (33)	Breast, cervical	Quasi-experimental intervention	Women with abnormal breast and cervical cancer screenings who were enrolled in the navigator intervention (*n* = 1,497) or usual care (*n* = 1,544) arm in the Boston Patient Navigation Research Program	There was a significant decrease in time to diagnostic resolution for navigated group compared with usual care group among those with a cervical screening abnormality (aHR = 1.46; 95% CI = 1.1–1.9); and among those with a breast cancer screening abnormality that resolved after 60 days (aHR = 1.40; 95% CI = 1.1–1.9). There was no difference before 60 days
Charlot et al. (34)	Breast, cervical	Quasi-experimental intervention	Women with a breast (*n* = 655) or cervical (*n* = 602) cancer screening abnormality enrolled in the Boston Patient Navigation Research Program	Language concordance was associated with timelier diagnostic resolution for all women of the cervical cancer screening abnormality group during the first 90 days (aHR = 1.46; 95% CI = 1.18–1.80), but not after 90 days. Race concordance was associated with significant decreases in time to diagnostic resolution for minority women with breast and cervical cancer abnormalities
Donelan et al. (35)	Breast	Group comparison study	72 women with abnormal mammography enrolled in a navigator program. 181 women with abnormal mammography were in the non-navigated group	There was no difference in timeliness of care, preparation for the visit to the breast center, ease of access, quality of care, provider communication, unmet needs, and patient satisfaction between groups
Dudley et al. (36)	Breast	Quasi-experimental intervention	460 low-income Hispanic women (260 navigated, 200 usual care) with an abnormal breast cancer screening result or untreated biopsy in the University of Texas Patient Navigation Research Program	The average days from definitive diagnosis to initiation of therapy was significantly reduced overall with navigation (navigation vs. usual care, 57 vs. 74 days, *p* < 0.05)
Freund et al. (37)	Breast	Meta analyses	3,083 women with abnormal breast cancer screening tests and 1,455 women with abnormal cervical cancer screening tests who participated in the Patient Navigation Research Program	One out of seven sites focused on abnormal breast cancer screening and two out of four sites focused on abnormal cervical cancer screening reported a significant benefit of PN on diagnostic resolution after cancer screening abnormality from 0 to 90 days
				Three out of seven sites focused on abnormal breast cancer screening and 2 out of four sites focused on abnormal cervical cancer screening reported a significant benefit of PN during 91–365 days
Hoffman et al. (38)	Breast	Prospective, pre-post study	2,601 women (1,047 navigated, 1,554 usual care) with abnormal breast cancer screening result/clinical abnormality enrolled in the DC City-wide Patient Navigation Research Program	The average number of days to diagnostic resolution was significantly shorter for navigated women than non-navigated women (25.1 vs. 42.1 days, respectively, *p* < 0.001), particularly among women who had a biopsy (*p* < 0.001)
Lee et al. (39)	Breast	Controlled cluster randomized trial design	1,039 (494 navigated, 545 usual care) women with abnormal breast cancer screening result/clinical abnormality enrolled in the Moffitt Patient Navigation Research Program	Patient navigation did not increase the timeliness of diagnostic resolution during the initial 3 months of follow-up but started to reduce time to diagnostic resolution after 3 months (aHR = 2.8, 95% CI = 1.30–6.13) and had a significant effect after 4.7 months (*p* < 0.05)
Luckett et al. (40)	Cervical, vulvar	Descriptive study	4,199 women at a tertiary care referral colposcopy center implementing a patient navigator program to reduce non-show rates	No-show rates declined from 49.7 to 29.5% after implementation of the patient navigator program
Markossian et al. (41)	Breast, cervical	Quasi-experimental intervention	Underserved women with abnormal breast or cervical screening test results were assigned to either patient navigation intervention (*n* = 355) (the Chicago Cancer Navigation Project) or usual care groups (*n* = 413)	Compared with the usual care group, the breast navigation group had shorter time to diagnostic resolution (aHR = 1.65, 95% CI = 1.20–2.28) and the cervical navigation group had shorter time to diagnostic resolution for those who resolved after 30 days (aHR = 2.31, 95% CI = 1.75–3.06), with no difference before 30 days
Paskett et al. (42)	Cervical	Meta-analysis	2,317 women with low and high-risk cervical abnormalities from four Patient Navigation Program centers who received patient navigation (*n* = 1332) or usual care (*n* = 985)	Low-risk women in the navigated group showed improvement in timely diagnostic follow-up in all racial groups, but significant effects were only observed in non-English speaking Hispanic women (OR = 5.88, 95% CI = 2.81–12.29). No effect was observed in high-risk women
Percac-Lima et al. (43)	Cervical	Quasi-experimental intervention	533 Latina women with an abnormal Pap smear requiring colposcopy received patient navigation. The comparison group was 253 non-navigated Latinas with an abnormal Pap smear requiring colposcopy	Navigated women had significantly fewer missed colposcopy appointments over time, with the average falling from 19.8 to 15.7% (*p* < 0.05), compared with an insignificant increase in the no-show rates from 18.6 to 20.6% in the comparison group
Raich et al. (44)	Breast	Randomized clinical trial	628 patients with abnormal breast screenings tests randomized to either intervention (*n* = 308) or usual care (*n* = 320) arms in the Denver Patient Navigation Research Program	For the abnormal breast screening group, 92% of the navigated patients reached diagnostic resolution of the initial abnormal test, as compared with 77% for the usual care patients (*p* < 0.001)
Ramirez et al. (45)	Breast	Prospective, pre-post study	425 Latina women with abnormal breast cancer screening results enrolled in either a patient navigator program (Six Cities Patient Navigation Study) (*n* = 217) or usual care (*n* = 208)	The time to diagnosis was shorter in the navigated group (mean, 32.5 vs. 44.6 days in the usual care group; HR = 1.32). Navigation significantly shortened the time to diagnosis among women who had BI-RADS-3 radiologic abnormalities (mean, 21.3 vs. 63.0 days; HR = 2.42); but not among those who had BI-RADS-4 or 5 (mean, 37.6 vs. 36.9 days; HR = 0.98)
**PATIENT NAVIGATION AFTER PRIMARY DIAGNOSIS**				
Chen et al. (46)	Breast	Pre-post design, quasi-experimental intervention	100 newly diagnosed women with breast cancer who were enrolled in a navigator program (*n* = 51) and non-navigated (*n* = 49)	Overall adherence to the quality indicators significantly improved from 69 to 86% (*p* < 0.01) with the use of patient navigators. Only one individual indicator, use of surveillance mammography, significantly improved (52–76%, *p* < 0.05) for the navigated women, not for the non-navigated women
Haideri and Moormeier (47)	Breast	Retrospective case series analysis	157 women who received navigation services and 103 women who received usual care after being diagnosed with breast cancer	There was no difference in the stage of presentation or the overall survival between the intervention and usual care groups. For the navigated women, there was a modest decrease (9 days) in the time between initial presentation and definitive therapy
Hendren et al. (48)	Breast	Randomized controlled trial	319 newly diagnosed breast cancer patients were randomized to receive a patient navigation intervention for improved quality of life (*n* = 141) or usual care (*n* = 129) in the University of Rochester Patient Navigation Project	There was no significant effect of patient navigation on disease-specific quality of life scores between navigated and usual care breast cancer patients undergoing primary cancer treatment
Ko et al. (49)	Breast	Multisite, quasi-experimental intervention	1,004 (navigated = 498, usual care = 506) women newly diagnosed with breast cancer enrolled in the Patient Navigation Research Program to improve receipt of recommended care	Among women eligible for antiestrogen therapy, navigated women had a significant higher likelihood of receiving antiestrogen therapy compared with non-navigated controls (OR = 1.73, *p* < 0.01). Among the women eligible for radiation therapy after lumpectomy, navigated women were no more likely to receive radiation than women in the usual care group (OR = 1.42, *p* = 0.22)
Madore et al. (50)	Breast	Quasi-experimental pilot study	20 medically underserved women recently diagnosed with breast cancer who were enrolled in the Breast CARES intervention to overcome treatment barriers	There was a decrease in depression and cancer-related distress and an increase in social support. Participation in the intervention helped the women overcome financial barriers (73.0%), transportation problems (60.0%), and communication barriers with medical staff (73.0%)
Raj et al. (51)	Breast	Retrospective, pre-post study	186 women with breast cancer from a disadvantaged minority community who participated in a patient navigator program to improve quality measures	Women who received navigation services received high-quality cancer care, as defined by concordance with ASCO/NCCN quality measures. These navigated women also had a favorable breast cancer stage distribution with >50% having *in situ* or stage 1 disease
Ramirez et al. (52)	Breast	Quasi-experimental intervention	480 Latinas with breast cancer enrolled in either a patient navigation program for timely diagnostic resolution (*n* = 251) or usual care (*n* = 229) in the Six Cities Study	A significantly higher percentage of navigated women initiated treatment within 30 days (69.0 vs. 46.3%, *p* < 0.05) and 60 days (97.6 vs. 73.1%, *p* < 0.001) compared to women in the usual care group. Time from cancer diagnosis to first treatment was significantly lower in the navigated group (22.22 days) than usual care group (48.30 days)
Ulloa et al. (53)	Breast	Prospective, pre-post study	130 low-income women from California enrolled in a patient navigation intervention to improve communication about survivorship care	The intervention significantly improved short-term recall of patient-specific breast cancer knowledge (*p* = 0.05) and reduced communication barriers (15.0% at week 1 to 6% at 3 months, *p* < 0.05)

## Results

### PN for Cancer Screening

The literature on PN interventions to increase breast cancer screening included five studies (26–30), with two randomized controlled trials (RCTs) (27, 30). In a large RCT with 3,895 inner city women, Phillips et al. (30) found no statistical difference in mammography adherence between the control (usual care) and PN intervention groups at baseline. After the 9-month intervention, mammogram adherence was significantly higher in the PN intervention group compared to the control group (87 vs. 76%, respectively, *p* < 0.001). Marshall et al. (27) implemented a RCT to increase breast cancer screening among 1,905 older African American Medicare beneficiaries. Women in the intervention group who received educational materials and PN services had significantly higher odds of being within guidelines for mammography screening at the end of the 2-year follow-up period compared to women in the control group who received only educational material [odds ratio (OR) = 2.26, 95% confidence interval (CI) = 1.59–3.22].

The other three studies (26, 28, 29) examined the effectiveness of PN interventions to increase breast cancer screening among diverse populations, including African American, Latina, Native American (26), immigrant (28, 29), and refugee (28, 29) women. Burhansstipanov et al. (26) implemented an education-based PN intervention to facilitate mammography screening for African American, Latina, Native American, and poor white women in the Greater Denver Metropolitan area. Statistically significant associations were found between having received the PN intervention and reporting a mammogram screening for all racial/ethnic groups (*p* < 0.05). A study by Percac-Lima et al. (28) implemented an educational, language concordant PN program for Serbo-Croatian refugees and immigrants to overcome barriers to breast cancer screening and support them in scheduling a mammogram. They found that, at baseline, 44% of women had a mammogram within the previous year, with the proportion increasing to 67% after 1-year (*p* = 0.001) of receiving the education-based PN intervention. Lastly, another study by Percac-Lima et al. (29) found that an education-based PN intervention to overcome barriers to breast cancer screening and information on how to obtain mammogram screening when needed among Somali, Arabic, or Serbo-Croatian refugee women improved mammography rates and significantly decreased disparities in screening rates between refugee and English- and Spanish-speaking women receiving care at the same health center.

One study in our review examined the impact of PN on screening rates for gynecological cancers. Wang et al. (31) found Chinese women in need of a Pap test reported significantly higher Pap test screening rates for those who received the PN intervention (education and PN services) compared to the control group (education only) (70 vs. 11.1%, respectively, *p* < 0.001).

### PN for Diagnostic Resolution

Six studies (32, 35, 36, 38, 39, 45) were identified that focused on PN interventions to reduce time from abnormal breast cancer screening to diagnostic resolution. Of the six studies, one RCT by Lee et al. (39) examined the efficacy of PN among medically underserved populations in Tampa, FL, USA. Results showed a lagged effect of PN; PN did not increase the timeliness of diagnostic resolution during the initial 3 months of follow-up [adjusted hazard ratio (aHR) = 0.85, 95% CI = 0.64–1.13], but reduced the time to diagnostic resolution after 3 months (aHR = 2.8395% CI = 1.30–6.13) and had a significant effect (*p* < 0.05) after 4.7 months. Several quasi-experimental studies (32, 36, 38, 45) on PN and diagnostic resolution for abnormal breast cancer screening reported that PN significantly shortened time to diagnostic resolution compared to women who did not receive PN. One cohort study (35) exploring patient perspectives of clinical care and PN in follow-up of abnormal mammography reported no differences in the timeliness of care, preparation for the visit to the breast center, ease of access, quality of care, provider communication, unmet needs, and patient satisfaction between navigated and non-navigated groups.

Three studies, a meta-analysis (42), descriptive study (40), and quasi-experimental study (43), were published during the time period reviewed that examined PN for diagnostic resolution of an abnormal cervical cancer screening result. A recent meta-analysis by Paskett et al. (42) examined the effectiveness of PN for diagnostic resolution of an abnormal cervical cancer screening among four PNRP centers. Within these centers, low-risk women in the navigated group showed improvement in timely diagnostic follow-up in all racial groups, but statistically significant effects were only observed in non-English speaking Hispanic women (OR = 5.88, 95% CI = 2.81–12.29). No effect was observed in high-risk women. A pre-post study (40) implemented a PN program to reduce no-show rates at a colposcopy center. After implementation, no-show rates for abnormal Pap test follow-up declined from 49.7 to 29.5% (*p* < 0.0001). In another quasi-experimental study (43) focused on Latinas in need of abnormal Pap test follow-up, navigated women had significantly fewer missed colposcopy appointments over time (i.e., reduction from 19.8 to 15.7%; *p* = 0.02) compared with an insignificant increase in no-show rates from 18.6 to 20.6% in the comparison group.

Three studies (33, 34, 41) explored PN interventions with regard to diagnostic resolution after an abnormal breast or cervical cancer screening test. A study by Markossian et al. (41) reported PN for abnormal breast cancer screening was associated with shorter time to diagnostic resolution (aHR = 1.65, 95% CI = 1.20–2.28, *p* = 0.002). However, there was a lag in the effectiveness of PN regarding diagnostic resolution for abnormal cervical cancer screening. In the first 30 days, the difference between those in the PN arm vs. those in the comparison group was not significant. But, from days 31 to 365, women in the PN group experienced a shorter time to diagnostic resolution compared with those women who received usual care (aHR = 2.31, 95% CI = 1.75–3.06, *p* < 0.001). A similar trend was noted by Battaglia et al. (33) among participants with an abnormal breast and cervical cancer screening test. Conversely, Charlot et al. (34) found a language concordance PN intervention was associated with timelier resolution for the cervical cancer screening abnormalities group during the first 90 days (aHR = 1.46, 95% CI = 1.18–1.80), but not after 90 days. No significant difference was found between the navigated and non-navigated breast cancer screening abnormality groups throughout the course of the study.

The PNRP studies included other cancers (colorectal and prostate), but the majority of the cancers were breast and cervical. A meta-analysis by Freund et al. (37) assessed the timeliness of diagnostic resolution for an abnormal breast and cervical cancer screening result across the PNRP. The results of the meta-analysis found little benefit during the first 90 days of care as only one of the seven sites focusing on breast cancer screening and two of the four sites focusing on cervical cancer screening observed a positive effect of PN on time to diagnostic resolution (*p* < 0.05). Greater benefit from navigation was seen from 91 to 365 days for diagnostic resolution among three of the seven sites focused on breast cancer screening and two of the four sites focused on cervical cancer screening (*p* < 0.05). Meta-regression revealed that navigation had its greatest benefits within centers with the greatest delays in follow-up under usual care.

One study (44) reported on the difference in time to diagnostic resolution between those in the PN intervention vs. control groups. A RCT from the Denver PNRP center evaluated the effectiveness of PN programs for increasing rates of diagnostic resolution for abnormal breast cancer screening. Raich et al. (44) found PN shortened time to resolution in the navigated group (*p* < 0.001) compared to the usual care group. Specifically, PN improved diagnostic resolution for patients presenting with mammographic BIRADS 0 and 3, but not BIRADS 4/5 or abnormal breast examinations.

### PN after Diagnosis

The results of the literature review for PN after cancer diagnosis resulted in eight studies (46–53), including one RCT (48), reporting effects on various outcomes among cancer patients including start of treatment, receipt of recommended care, completion of treatment, quality of life and depressive symptoms, communication with physicians, and quality measures. Hendren et al. (48) found no significant effect of PN on disease-specific quality of life scores between navigated and usual care breast cancer patients from baseline to 3 months. Other studies suggest that PN had no effect on time to completion of primary cancer treatment, satisfaction with cancer-related care, or psychological distress, and they attributed the non-significant findings to the open eligibility criteria (all patients) instead of targeting those with shown need, as seen in other effective interventions (49, 51, 52).

A study by Ramirez et al. (52) sought to examine the effectiveness of PN in reducing time from breast cancer diagnosis to initiation of treatment among Hispanic/Latino women. Compared to control patients, there was a significantly higher percentage of navigated women who initiated treatment within 30 days (69.0 vs. 46.3%, *p* < 0.05, intervention vs. control, respectively) and 60 days (97.6 vs. 73.1%, *p* < 0.001, intervention vs. control, respectively) from diagnosis. Also, time from breast cancer diagnosis to first treatment was significantly lower in the navigated group (22.22 days) than among women in the control group (48.30 days).

In a large, multisite study, Ko et al. (49) sought to improve the receipt of recommended care for newly diagnosed breast cancer patients, and the findings varied based on the type of treatment received by the patients. Among women eligible for antiestrogen therapy, navigated participants were more likely to receive antiestrogen therapy compared with usual care participants (OR = 1.73, *p* = 0.004). Among women eligible for radiation therapy after lumpectomy, navigated participants were no more likely to receive radiation than usual care participants (OR = 1.42, *p* = 0.22).

### Barriers to Care and PN

Several studies have conducted secondary analyses to understand the association between barriers to care and clinical outcomes, particularly within the PNRP. A 2015 study by Ramachandran et al. (54) explored the association among number of barriers to care, type of barriers, and timeliness of diagnostic resolution among women with abnormal cancer screening results. They found that 74% of breast cancer screening participants and 55% of cervical cancer screening participants reported at least one barrier to diagnostic resolution. Navigated women with barriers resolved cancer screening abnormalities at a slower rate compared with navigated women with no barriers. Another study by Ramachandran et al. (55) using Boston PNRP data, found the odds of timely diagnostic resolution reduced as the number of barriers increased (one barrier, aHR = 0.81, 95% CI = 0.56–1.17, *p* = 0.26; two barriers, aHR = 0.55, 95% CI = 0.37–0.81, *p* = 0.0025; three or more barriers, aHR = 0.31, 95% CI = 0.21–0.46, *p* < 0.0001). Lastly, Katz et al. (56) examined the effect of having barriers to diagnostic resolution and time to resolution among participants in the PN intervention arm with a breast or cervical cancer abnormality in the PNRP. They found that 63.7% of breast abnormality and 46.6% of cervical abnormality participants had at least one barrier resulting in longer time to diagnostic resolution among breast (aHR = 0.74, 95% CI = 0.67–0.83, *p* < 0.01) and cervical (aHR = 0.79, 95% CI = 0.70–0.90, *p* < 0.01) participants vs. those with no reported barriers.

Specific types of barriers patients report were described by several studies (57–59). Korber et al. (57) found the most common barriers to cancer treatment were patient-provider communication and knowledge of patient resources. Other studies found location of health-care facility (59), transportation problems (58), not speaking English (55), no insurance (56), financial concerns (58, 60), lack of social/practical support (58, 59), and lack of information about the abnormality (57) as the most prevalent barriers to cancer care among patients enrolled in PN interventions.

Some studies have attempted to determine which variables are associated with having a barrier to cancer care to identify women most in need of PN. Several studies (55, 60–62) found that women with barriers to cancer care were more likely to be racial and ethnic minorities (55, 60), unmarried (62), part-time employed/unemployed (60, 62), non-English language speakers (55, 61), and have public/no health insurance (55, 60) compared to women without any barriers to care.

## Discussion

As evidenced in this literature review, PN has been shown to help women receive cancer screenings, receive more timely diagnostic resolution after a breast and cervical cancer screening abnormality, initiate treatment sooner, receive proper treatment, and improve quality of life among cancer patients. Also, it was shown that PN eliminates barriers to care. PNRP demonstrated: (1) who has barriers; (2) that barriers delay the receipt of care; and (3) types of numbers of barriers that impact time to treatment (54–56).

Several trends emerged from this review. PN programs have been implemented among diverse populations specifically focused on reducing health disparities in racial and ethnic minorities and/or underserved populations. It is important to note that each study population and setting was unique, thus generalizability of these findings may be limited. Another trend noted was the limited effectiveness for certain groups receiving PN, alluding to the possibility that PN is not equally effective for all groups. Results found significant differences in PN effectiveness with regard to age (32), ethnicity (42), location of care (37), type of screening test (38), and type of treatment (49). Although relevant for all populations, use of a “one-size-fits all” approach to PN may not be the best approach. The original intent of PN is to improve the experience of care among patients with the greatest needs by tailoring actions to an individual’s barriers to care (63). If implementation across the patient population is too demanding on resources, especially due to the fact that the American College of Surgeons Commission on Cancer (CoC)’s accreditation mandates are currently unfunded, targeting PN implementation may be a possible solution. By identifying those most likely to need PN, scarce resources can be diverted to women in most need and most likely to delay or not receive prompt, appropriate care (64).

Finally, although there is evidence of the potential of PN to improve outcomes related to cancer screening and diagnostic resolution, many studies have utilized less robust designs (i.e., quasi-experimental and descriptive studies), as mentioned by previous reviews (14–16). A notable difference between this review and the prior reviews is an increase in the number of studies evaluating PN on cancer screening and treatment outcomes. Another difference between this and previous reviews was inclusion of studies that evaluated the association of PN with reported outcomes during cancer treatment and post-treatment. Yet, there was great heterogeneity among the studied outcomes (e.g., quality of life, proper treatment), and therefore, cumulative evidence, as seen in PN interventions on cancer screening and diagnostic resolution, is lacking.

### Research Opportunities in PN and Women’s Health

Due to the increased prevalence of PN in health-care systems, there are growing research opportunities in PN and women’s health. One area that is ripe for researchers is PN in cancer survivorship, particularly among post-treatment cancer survivors. Increases in the number of individuals diagnosed with cancer each year, as well as improving survival rates, have led to an ever-increasing number of cancer survivors (20). As evidenced by this review, implementation of PN interventions among post-treatment cancer survivors is lacking. Future research should not only explore adherence to post-treatment surveillance behaviors but also treatment outcomes that can affect the physical and psychological well-being of women.

The PN literature on women’s cancers is growing; however, it is limited in that researchers have primarily focused on breast cancer (as seen in the literature on PN after diagnosis). Although cervical cancer incidence and mortality rates have steadily decreased, it is estimated that in 2015, 12,900 new cases of invasive cervical cancer occurred and 4,100 died from this disease (19). There is a wide racial and socioeconomic disparity in the incidence and mortality rates from cervical cancer. Underlying these disparities are often education, language, geographic, and trust factors (65). As evidenced by this review, the few studies that have explored PN in gynecological cancers showed promising results. Thus, researchers should make gynecological cancers a focus for their PN interventions to maximize the positive impact on this survivor population. Important yet understudied subpopulations, such as women with increased genetic risk, should be considered. PN can provide education, support, and guidance within the clinical setting for these women to receive appropriate screenings, genetic testing and counseling, prompt diagnosis, and proper treatment.

Women may also benefit from PN during cancer care that is tailored to specific family-related barriers, such as child care and transportation. Women often assume the role of caregivers and income-earners and may need more assistance in caregiving for others while receiving cancer care for themselves. PN can link them to resources that offer emotional (i.e., support groups) and tangible support (i.e., house cleaning, child care) and has the potential to improve quality of life and psychosocial outcomes for both women with cancer and women who are caregivers.

Navigators should consider the age of the female patient and their stage in life during PN interventions. For example, younger women often face very different challenges and complications than older women, including concerns about becoming a mother; caring for children when faced with a life-threatening illness; premature menopause leading to loss of fertility; sudden onset of vasomotor symptoms; long-term consequences of early ovarian decline; body image and sexuality; and career and work concerns related to productivity and job security (66). For older adults, age-related concerns may include spousal caregiving; lack of social support; quality vs. quantity of life; comorbidities; risk of polypharmacy; mobility challenges; housing and transportation needs; and declining cognitive function and information processing (67, 68). Navigators from the PNRP used a standardized, structured list and an open-ended approach that captured barriers to care identified by participants. Future studies should utilize this approach to collect information on barriers to care and explore age-related differences in reported barriers to address individual needs accordingly.

The similarity of patient navigators to the participants is important for the success of PN interventions. For example, patient navigator race and language concordance improved the timeliness of care in a minority population (34). Likeness to the patient population is already a typical characteristic of CHWs, who work within a target community to improve community awareness and adherence to cancer screenings, and thus, this successful strategy should be extended to navigators. Since the CHWs’ role is to connect underserved populations from the community with screening services, PN programs should work with CHWs to assist women across the entire cancer care spectrum from cancer prevention to post-treatment. Future research should explore the effect of a combined CHW and PN intervention to increase community engagement, improve access to preventive health services, facilitate timely diagnosis and treatment, and ultimately improve the health of women in underserved areas.

## Author Contributions

JK-S was responsible for the planning, literature review, writing, editing, and submitting the manuscript. JO was responsible for the planning, literature review, writing, and editing the manuscript. EP was responsible for the planning, writing, and editing the manuscript.

## Conflict of Interest Statement

The authors declare that the research was conducted in the absence of any commercial or financial relationships that could be construed as a potential conflict of interest.
